# A Novel Method for Identifying Patients with High Risk of Litigation in Plastic Surgery: Introducing the FATIMA Acronym

**DOI:** 10.1007/s00266-024-04476-2

**Published:** 2024-10-30

**Authors:** Miriam Vicente-Ruiz, Bernardo Hontanilla

**Affiliations:** Department of Plastic and Reconstructive Surgery, Clínica Universidad de Navarra, Av. Pio XII 36, 31008 Pamplona, Spain

**Keywords:** Litigation, Plastic surgery, Medical liability

## Abstract

**Background:**

In recent years, there has been an upward tendency in the number of claims against Plastic Surgeons in some countries, while remaining among the most litigated specialists worldwide. Identifying the most frequent traits in Plastic Surgery claimants could aid surgeons in performing better patient selection and avoid liability.

**Methods:**

The three main legal databases in Spain were consulted for Plastic Surgery litigations in Spain over a five-year period. Data from the plaintiffs, defendants and judicial processes were collected. In addition, expert witnesses were interviewed and data from the main medico-legal association in Spain was collected. This data was contrasted with the available literature regarding litigation in Plastic Surgery worldwide.

**Results:**

A total of 199 court resolutions were analyzed, two expert witnesses were interviewed and “The Patient’s Advocate Association” of Spain was consulted. The most frequent traits observed in the plaintiffs were summarized in the acronym FATIMA that describes a Female patient using Antidepressants or Anxiolytics, presenting body Tattoos, suing after undergoing a breast surgery with the use of Implants, Middle-aged and with Access to free legal services.

**Conclusions:**

The use of the acronym FATIMA in the identification of patients with high risk of litigation in Plastic Surgery could aid surgeons in performing adequate patient selection and avoiding medical liability.

**Level of Evidence IV:**

This journal requires that authors assign a level of evidence to each article. For a full description of these Evidence-Based Medicine ratings, please refer to the Table of Contents or the online Instructions to Authors  www.springer.com/00266.

## Introduction

In recent years, countries like Spain, Italy, and Canada have seen an upward trend in the number of claims against plastic surgeons, while other countries, such as the USA and Japan, have experienced stability or even a slight decline in these numbers [1–6]. Despite this, plastic surgeons continue to be among the most litigated specialists globally. In Europe, they rank among the top three most litigated specialties in countries like Italy and Spain, whereas in the USA and Taiwan, they fall between the fourth and sixth positions [1, 2, 6–8]. In the USA, plastic surgeons have the second highest rate of paid claims per physician, second only to neurosurgery, accounting for 1.75% of all medical payments [5, 6]. In South Korea, plastic surgeons are responsible for 7% of total payments, placing them third after orthopedics and internal medicine [9].

To reduce the likelihood of a lawsuit, strategies include maintaining excellent communication with patients, engaging in shared decision-making, and ensuring thorough and accurate medical records [5]. Additionally, recognizing common traits among plaintiffs in plastic surgery cases could aid surgeons in performing adequate patient selection as well as exercising caution when faced with patients with high risk of litigation.

To identify the key characteristics of litigants in our specialty, we analyzed the outcomes of plastic surgery lawsuits in Spain over a five-year period, gathering data from expert witnesses and medico-legal associations. As a result, we have developed a new acronym for identifying patients with a high risk of litigation.

## Methods

In order to collect the court’s resolutions related to plastic surgeries in Spain from January 1, 2018, to December 31, 2022, the three main national legal databases were consulted: CENDOJ, ARANZADI and VLEX [10–12]. These databases are public primary sources used by legal professionals and contain no protected patient information. The following search terms were used: “plastic surgery”, “aesthetic surgery” and “reconstructive surgery”. The verdicts were collected and revised manually, and only those in which a plastic surgery procedure was the primary reason for the litigation were included. The following data were extracted: date, claim type, claimant and defendant, procedure involved, reason for the claim, damage claimed, outcome and compensation.

To gather further data about the characteristics of litigants in Spain, we interviewed two Plastic Surgeons that serve as expert witnesses in trials. We specifically inquired after the information that is not accessible to the public such as the age or medical history, as well as other remarkable details. In addition, we consulted the 2018 to 2022 Annual Reports of the “Patient’s Advocate Association”, the main Spanish association to provide legal assistance for patients that have been affected by any type of medical malpractice, and we collected the information relative to claims caused by Plastic Surgery procedures [13].

Lastly, we performed a literature review for Plastic Surgery lawsuits worldwide, looking for characteristics about the claimants as well as information about litigation in Plastic Surgery in other countries.

## Results

2020 court resolutions were collected from the three databases. After exclusion of duplicates and manual revision, 199 resolutions relative to plastic surgeries were included (Table 1). In Spain the sentences from the Courts of First Instance are not published unless they are related to a landmark or notable ruling, so they are generally inaccessible to the public. The collected verdicts therefore belong to higher instance courts that have received the cases after the plaintiff or the defendant has appealed the first instance court’s decision.

**Table 1:** Data extracted from the court resolutions related to plastic surgery litigations in Spain from 2018 to 2022

N (%)	
*Year*	
2018 2019 2020 2021 2022	45 (23) 25 (12) 35 (17) 53 (27) 41 (21)
*Court*	
Civil Administrative Penal	157 (79) 30 (15) 12 (6)
*Plaintiffs*	
Women Men Not specified	189 (95) 8 (4) 2 (1)
*Defendants*	
Male Surgeons Female Surgeons	163 (82) 36 (18)
*Specialty of defendants*	
Not specified Plastic surgeons Other specialties	124 (62) 65 (33) 10 (5)
*Surgical field*	
Breast Body countouring Facial Hand Other	136 (68) 58 (29) 28 (14) 3 (2) 3 (2)
*Most frequent procedures involved*	
Augmentation Mammoplasty Augmentation Mastopexy Abdominoplasty Liposuction Breast Reduction Implant Replacement Rhinoplasty Blepharoplasty	61 (31) 37 (19) 28 (14) 23 (12) 10 (5) 10 (5) 10 (5) 10 (5)
*Type of surgery:*	
Cosmetic Reconstructive	167 (84) 32 (16)
*Reason for the claim*	
Unsatisfactory result Lack of information Secondary injuries Surgical complications Diagnostic errors Death	147 (74) 107 (54) 6 (3) 2 (1) 2 (1) 1 (1)
*Damages claimed*	
Secondary surgeries Surgical complications Unesthetic result Death	128 (64) 107 (54) 85 (43) 2 (1)
*Outcome of the claim*	
Acquittal Conviction Inadmission	108 (54) 82 (41) 9 (5)
*Reason for conviction*	
Faulty informed consent Lack of information Malpractice Negligence Imprudence Contract liability Non-delivered result Abandonment of care Disproportionate damage	21 (25) 21 (25) 20 (24) 12 (14) 7 (8) 4 (5) 4 (5) 3 (4) 1 (1)
*Compensation*	
Average Maximum Minimum	30,615 EUR (33,165 USD) 304,042 EUR (329,373 USD) 1,600 EUR (1,733 USD)

We observed a mild reduction of resolutions in 2019 and 2020 probably due to the pandemic that was later compensated with an increase in 2021. The majority belonged to Civil Courts, followed by Administrative and Penal Courts. An overwhelming predominance of women was observed among the plaintiffs, while most defendants were male surgeons. Regarding the specialty of the claimed surgeons, in one-third of the cases, it was a Plastic Surgeon, whereas in two thirds it was not specified and, in a minority of cases, it was a different specialist such as a General Surgeon, an Ear-Nose-Throat Surgeon or an Ophthalmologist. The procedures involved were predominantly breast surgeries, followed by body contouring and facial surgeries. The most frequent surgeries involved were augmentation mammoplasty in one third of the cases and augmentation mastopexy in one-fifth, followed by abdominoplasty and liposuction. A minority were due to breast reductions, implant replacements, rhinoplasties and blepharoplasties. These surgeries correspond with the most frequently performed cosmetic surgeries in Spain, with breast augmentation, mastopexy, abdominoplasty and liposuction among the five most performed interventions in this same period [14]. As expected, most of the lawsuits were due to cosmetic surgeries, as opposed to reconstructive surgeries.

The main reasons for the lawsuit were predominantly an unsatisfactory result of the surgery, followed by a lack of information. Other reasons included secondary injuries, surgical complications or diagnostic errors. Lawsuits frequently included more than one reason. The damages claimed included surgical complications in more than half of the cases with most of the claimants needing to undergo secondary correction surgeries. Of these, 42% underwent a single secondary surgery and 22%, two or more. In addition, four out of ten patients reported an unesthetic result. Only two resulted in the death of the patient.

The court’s decision included acquitting of the defendant in more than half of the cases and conviction in four out of ten, with very few resulting in inadmission. From the 82 that resulted in conviction, the reasons for the court’s decision included: a faulty informed consent or a lack of information, followed by malpractice due to a lack of experience or poor execution of the technique. Negligence and imprudence were also noted, while a minority were due to contractual liability, a non-delivered result that had been guaranteed, abandonment of care or a disproportionate damage. As for compensation, the average pay was 30,615 euros (33,165 USD) with a maximum of 304,042 (329,373 USD) and a minimum of 1600 euros (1733 USD). There was only one criminal conviction corresponding to 1.5 years in prison and a 304,042-euro compensation.

Because of confidentiality, the two expert witnesses could not share individual data about their clients. However, they provided approximate information about the cases they had been involved in during the period of 2018 to 2022, with a total number of 450 reports between the two experts (Table 2). They were both interviewed separately, one in person and one telephonically. According to their declarations, more than 90 percent of the plaintiffs were women with an average age between 30 and 40 years. As relevant medical history, approximately 75 percent of them presented some underlying mental pathology, especially anxiety-depressive disorder, and routinely took anxiolytic or antidepressant drugs. As remarkable features, many of them had body modifications such as tattoos. Finally, most of them had been treated in low-cost clinics or medical spas, having undergone breast procedures with the use of implants, and often having resorted to bank loans to be able to afford them. In addition, many of them had access to free legal services and their demands were aimed at obtaining a financial benefit because of some complication of the surgery or an unesthetic result. In Spain, free legal services are offered to people who lack the necessary financial means to cover the expenses derived from a lawsuit.

**Table 2:** Declarations from the two expert witnesses

Questions	Expert witness 1	Expert witness 2
“How many cases have you been involved in between 2018 and 2022?”	“60 cases per year”	“30 cases per year”
“What percentage of plaintiffs were women?”	“Over 90 percent”	“Almost all of them”
“What was their average age?”	“Approximately 35-36 years”	“Between 30 and 40”
“Did they have any relevant medical history?”	“Approximately three out of four took psychotropic drugs, mainly antidepressants and anxiolytics”	“Many of them suffered from either depression or anxiety and took medication for that”
“Did they have any substance abuse?”	“Some of them were smokers but less than fifty percent”	“Very rarely, mostly tobacco”
“Did you notice anything relevant in the clinical photographs?”	“Many times, you could see that they had some sort of tattoo”	“Surprisingly a lot of them had some body modification like a tattoo or piercings”
“What type of establishments were they treated in?”	“A lot of them came from a low-income background so they resorted to low-cost establishments and often asked for loans or deferred payments”	“Low-cost clinics and medical spas”
“What types of surgeries caused the lawsuits?”	“Surgeries for breast augmentation and mastopexy”	“Mostly breast aesthetic surgeries with implants”
“What was the reason for their claim?”	“They mostly complained of a bad result because of some complication”	“They were not happy with the result and often claimed that they weren’t adequately informed of the risks”
“What do you think their motivation was?”	“A lot of them had access to free legal assistance so everything was free of cost, they had nothing to lose and could get a lot of money out of it”	“Money mostly, as many have free legal services”

The “Patient’s Advocate Association” of Spain (Asociación “El Defensor del Paciente”) gave assistance to a total of 1,556 claims due to Plastic Surgeries from 2018 to 2022 [13]. In their 2023 Annual Report, they declare that a significant number of these claimants suffer from some mental health problem, so they insist on the importance of analyzing the mental state of patients by performing routine psychological tests before performing these procedures.

Based on the findings from the analyzed claims as well as the information provided by the experts and the “Patient’s Advocate Association”, we have developed an acronym for identifying patients with high risk of litigation in Plastic Surgery. This acronym corresponds to the name: FATIMA (Table 3).

**Table 3:** A novel acronym for identifying patients with high risk of litigation in plastic surgery: FATIMA

F	Female
A	Antidepressants/Anxiolytics
T	Tattoo
I	Implants
M	Middle age
A	Access to free legal services

## Discussion

The use of acronyms to aid in the selection of patients in Plastic Surgery is not novel. In 1999, Gorney and Martello proposed the acronym SIMON, which summarized the typical traits of patients most likely to show dissatisfaction after a cosmetic surgery and consequently cause problems [15]. According to the authors, these features served to alert surgeons about the type of patients they should avoid operating. However, these characteristics have not been linked to higher litigation rates and include psychological traits that might be difficult to identify in a standard surgical consultation. From our findings, we consider the FATIMA acronym to be more accurate as it corresponds to the most frequent traits found in plaintiffs in our study and is in concordance with previously published literature as well as with the available information regarding claimants in Plastic Surgery in other countries. Furthermore, we consider that these features, collected for the first time in this acronym, are easy to identify and accessible to the surgeon from the first preoperative consultation.

### Female

Multiple studies have found that claims in Plastic Surgery come predominantly from female patients [5, 16, 17]. Women account for approximately 85 percent of cosmetic surgeries worldwide and these surgeries are the ones more frequently originating lawsuits [18]. However, their prevalence among claimants is proportionately higher accounting for more than 90 percent in most studies. In our study, where women represented 95 percent of lawsuits while they constituted 85 percent of all cosmetic surgeries in Spain during the same period. Conversely, a majority of plaintiff surgeons are male with one study showing a higher incidence in doctors over 55 years of age [16, 17, 19]. Once again, while male surgeons make up the majority of plastic surgeons in most countries, they are disproportionately represented among defendants [20]. In Spain, where men account for 63 percent of Plastic Surgeons, they constituted 82 percent of defendants in our study [21]. Evidence also suggests that patients tend to be more satisfied when treated by doctors of the same gender, which might contribute to the higher likelihood of female patients suing male surgeons [22].

### Antidepressants/Anxiolytics

The use of psychotropic medicines such as antidepressants and anxiolytics has increased in recent years with high-income countries such as the USA and Spain having high consumption rates [23]. Although the connection between mental health and litigation is not well-established, it is reasonable to consider that mental instability may lead to potential conflicts. Consequently, conducting psychological assessments beforehand could help plastic surgeons in selecting patients and provide evidence for defense in court if litigation arises.

### Tattoos

The expert witnesses described a high prevalence of tattoos among the plaintiffs, which could be linked to the patient’s psychological traits and socioeconomical status. Although tattoos have distinct implications in certain cultural settings, some studies suggest that people with tattoos have higher impulsivity scores and aggressiveness than the general population [24, 25]. Other studies also indicate a higher prevalence of tattoos among people with a lower educational and socioeconomic level [24, 26]. This could potentially increase the likelihood of conflict and subsequent litigation if misunderstandings arise between the patient and the surgeon.

### Implants

Breast surgeries with the use of implants, including augmentation mammoplasty and augmentation mastopexy, have been consistently found to be the most frequent causes of litigation in Plastic Surgery [1–3, 5, 16, 27]. While they are among the most frequently performed surgeries globally and their numbers are in rise, they cause a considerably larger proportion of claims if we compare it with other frequently performed procedures such as liposuction [18]. In effect, liposuction is the most popular cosmetic procedure worldwide and was previously second after augmentation mammoplasty, yet it has not been associated with high litigation rates and results in fewer claims compared to cosmetic breast surgeries [18].

### Middle Age

The median age of plaintiffs ranges between 30 and 40 in most studies, with an average age of 36 [5, 16, 28]. Considering that the biggest consumers of cosmetic surgery worldwide correspond to the age group of 35–50, closely followed by that of 18–34, the typical plaintiff would fall in between these two groups [18]. Middle-aged people typically have a higher level of awareness about their rights and the standard of care they should receive and may have higher expectations for positive outcomes as well as financial resources. As a result, they would be more likely and better equipped to pursue legal action.

### Access to Free Legal Services

In countries where free legal services are available, patients with access to these resources could have a higher motivation to litigate if a complication or an unesthetic result occurred. One potential method to identify these patients could be by examining the type of establishments they choose, as they are more likely to opt for low-cost clinics or seek a loan or deferred payment to afford the surgery.

Similar studies that analyze claims in Plastic Surgery lawsuits in different countries and legal settings yield similar results. Cosmetic surgeries consistently show higher litigation rates than reconstructive surgeries, while more than fifty percent of rulings are resolved in favor of the surgeon [1–3, 17, 29]. Also, lack of informed consent is the most frequently cited cause of condemnation [3, 29–31]. As for compensation amounts, they vary widely between countries with some studies indicating that the highest settlements are in the USA, averaging $171k, while other countries, like Canada ($60k), Italy ($50k), and Spain ($32k), have considerably lower payouts [1–3, 5].

It has been challenging to obtain comprehensive judicial information about the claims made against plastic surgeons in Spain, similar to other countries. This includes the impossibility to access data from first instance courts as well as protected information about claimants and defendants. Despite these limitations, we were able to gather this information from expert witnesses and medico-legal organizations and draw relevant conclusions.

Most authors agree that the majority of lawsuits in Plastic Surgery are not a result of technical failures, but they are rather due to inadequate patient selection and poor communication between the surgeon and the patient [5, 29, 32, 33]. In this sense, we hope that this acronym will aid surgeons in performing a better selection of patients and avoid litigation. Further studies will be necessary to elucidate the utility of this acronym and its application in different countries and legal settings.

## Conclusions

The use of the acronym FATIMA in the identification of patients with high risk of litigation in Plastic Surgery could aid surgeons in performing adequate patient selection and avoid medical liability. If a patient meets these criteria, it is up to the surgeon to discern the suitability of performing the surgery in each specific situation. In any case, the identification of this patient profile can put the surgeon on notice to exercise extreme caution and use all means to avoid a possible lawsuit.
